# Impact of understory vegetation on soil carbon and nitrogen dynamic in aerially seeded *Pinus massoniana* plantations

**DOI:** 10.1371/journal.pone.0191952

**Published:** 2018-01-29

**Authors:** Ping Pan, Fang Zhao, Jinkui Ning, Ling Zhang, Xunzhi Ouyang, Hao Zang

**Affiliations:** 1 College of Forestry, Jiangxi Agricultural University, Nanchang, China; 2 College of Tourism and Territorial Resources, Jiujiang University, Jiujiang, China; RMIT University, AUSTRALIA

## Abstract

Understory vegetation plays a vital role in regulating soil carbon (C) and nitrogen (N) characteristics due to differences in plant functional traits. Different understory vegetation types have been reported following aerial seeding. While aerial seeding is common in areas with serious soil erosion, few studies have been conducted to investigate changes in soil C and N cycling as affected by understory vegetation in aerially seeded plantations. Here, we studied soil C and N characteristics under two naturally formed understory vegetation types (*Dicranopteris* and graminoid) in aerially seeded *Pinus massoniana* Lamb plantations. Across the two studied understory vegetation types, soil organic C was significantly correlated with all measured soil N variables, including total N, available N, microbial biomass N and water-soluble organic N, while microbial biomass C was correlated with all measured variables except soil organic C. *Dicranopteris* and graminoid differed in their effects on soil C and N process. Except water-soluble organic C, all the other C and N variables were higher in soils with graminoids. The higher levels of soil organic C, microbial biomass C, total N, available N, microbial biomass N and water-soluble organic N were consistent with the higher litter and root quality (C/N) of graminoid vegetation compared to *Dicranopteris*. Changes in soil C and N cycles might be impacted by understory vegetation types via differences in litter or root quality.

## Introduction

Soil carbon (C) and nitrogen (N) play important role in impacting soil capacity to maintain biological productivity [1, 2] and regulating atmospheric C and N compositions [3–5]. Among soil C and N components, soil microbial biomass C (MBC) and N (MBN) are labile constituents in soil organic C (SOC) and total N (TN) and indicators of changes in soil C and N pools [6]. In addition, both soil water-soluble organic C (WSOC) and N (WSON) are sensitive to land use, forest management, and habitat disturbance. These labile constituents of soil organic matter are considered to be important indicators of soil capacity in maintaining biological productivity [7, 8].

In forest ecosystems, understory vegetation can be an important factor impacting soil C and N processes [9] as has been documented by previous studies [10, 11]. While understory vegetation represents only a small portion of forest biomass, it plays an important role in maintaining biodiversity, ecosystem stability, and sustainable productivity of forest ecosystems [12]. However, the dependence of soil C and N processes on understory vegetation types are not thoroughly understood [13]. Studies have shown that understory vegetation types impact soil temperature and moisture [14], microbial richness and composition [15–18], and C/N ratios [11, 13, 19]. For example, Wu et al. [20] reported that removing understory vegetation from *Eucalyptus* forest decreased root biomass and soil organic matter input, and hence altered soil microbial community structure. Similarly, Wan et al. [21] evaluated the effect of the understory vegetation (*Dicranopteris dishotoma*) on the growth of subtropical forest canopy vegetation (*Eucalyptus*) and found decreased soil moisture, soil organic matter, and soil pH after understory vegetation was removed. So, it is critical to understand the effects of understory vegetation on soil C and N cycling, and further, potential effects on changes in atmospheric C and N compositions [22, 23], especially in areas with intensive reforestation and management of understory vegetation.

In the mid-20^th^ century, the southern part of Jiangxi Province, China, was a region with large-scale intensive soil erosion and increased runoff, especially the Xing’guo County area. To control runoff and soil erosion and restore forest vegetation, aerial seeding of *P*. *massoniana* has been widely implemented since the 1970s [24]. Aerial seeding plantations characterized by faster restoration played important role in improving the ecological environment of the region. Considering the importance of understory vegetation in aerially seeded plantations, their effects on soil element cycling process could not be ignored. In the process of forest development, two understory vegetation types (*Dicranopteris* and graminoid) have formed. As the root system of *Dicranopteris* consists of clustering rhizomes, it can grow on the surface of soil through horizontal spread, effectively reducing water loss and mitigating soil erosion [25]. The root systems of graminoids differ from those of *Dicranopteris*. In addition, the dominant graminoid species *Paspalum thunbergii* may have symbiotic relationship with N-fixing soil bacteria as its congeners, which will potentially impact litter C and N characteristics and soil C and N cycling [26]. However, the effect of understory vegetation on soil C and N cycling process in aerially seeded plantations has not been investigated.

Here, we conducted a study in Xing’guo County to understand how soil C and N cycling vary in aerially seeded *P*. *massoniana* plantations [24] that differ in understory vegetation types to investigate the following questions: 1) How are soil C and N components related to each other in aerially seeded *P*. *massoniana* plantations that differ in understory vegetation? 2) How do vegetation functional characteristics vary between *Dicranopteris* and graminoid dominated understories? 3) How do soil C and N characteristics vary among plantations with understories dominated by *Dicranopteris* vs. graminoids? The results will provide a scientific reference for the management of understory vegetation in aerially seeded *P*. *massoniana* plantations.

## Materials and methods

### Study area

Xing’guo County (115°01’~115°51’ E, 26°03’~26°42’ N) located in the central southern part of Jiangxi Province, subtropical China, is characterized by a mid-subtropical warm and humid climate. The mean annual temperature is 18.9°C and annual precipitation is 1,539 mm, with a distinct wet season from April to June [27]. The frost-free period lasts for 280–300 days. Soils are classified as Udic Ferralsols developed from granite weathering [28]. This area is rich in forest resources. Presently, the main forests here are evergreen broadleaf forests, *P*. *massoniana* forests, and *Cunninghamia lanceolata* plantations [27, 29]. The area of aerially seeded *P*. *massoniana* plantations is 64,000 ha, accounting for 29.5% of the county’s forest land area [27]. These plantations have understories dominated by either *Dicranopteris linearis* or *Paspalum thunbergii*, respectively, which were formed 10–15 years later after *P*. *massoniana* were sowed. The dominated proportion of *Dicranopteris* and graminoid vegetation were more than 90% and 85%, respectively.

### Plot establishment and soil sampling

**Fig 1:**
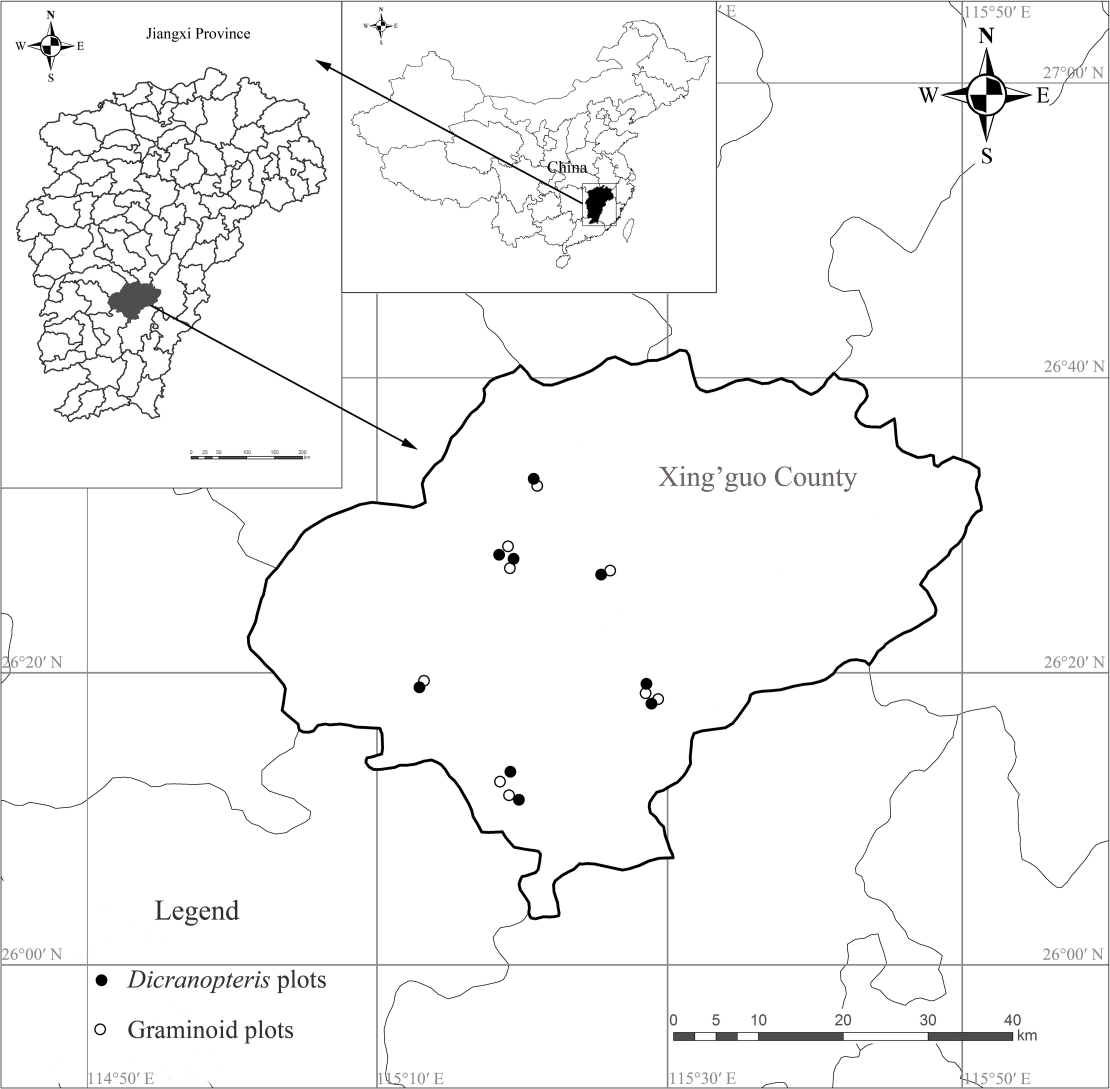
Sampling locations of the study area (Xing’guo County, Jiangxi province, China).

In August 2012, a reconnaissance survey was conducted in aerially seeded *P*. *massoniana* plantations. The plantations selected for this study had not been disturbed by anthropogenic activities since aerially seeding during 1986 and 1991, and had understories dominated by *Dicranopteris* or graminoid species (Fig 1). The slope faced south with an intermediate slope (S1 Table). The understory had a density of 60–80% cover. Plots were established paired and randomly in *P*. *massoniana* plantations with understory vegetation dominated by *Dicranopteris* or graminoid species (S1 Table; Fig 1). The plot size was 400 m^2^ (20 × 20 m), with nine replications for each understory vegetation type. No permits were required.

Soil samples were collected from the top 20 cm soil layer from three sampling points at the upper, middle, and lower part of each plot using a soil auger (6 cm in diameter). Soil samples of each plot were mixed together to obtain one composite sample. Approximately 1 kg of the mixed sample was then divided into two portions. One portion was preserved at a temperature of 4°C for the measurement of MBC, MBN, available N (AN), WSOC and WSON. The other portion was air-dried and processed for the determination of SOC and TN.

### Understory vegetation litter, root and biomass collection

Three randomly distributed subplots (1 m×1 m) were established for collection of litter, root and total biomass in each plot. In July 2012, plant litter and above- and below-ground biomass were each collected by vegetation types. Root collection was performed by excavating soils within the subplots. Specifically, subplot soil within the 0–30 cm soil layer was all collected and passed through 0.5 mm sieve to obtain all fine roots [30]. Soil attached to roots was removed by washing. Plant samples were taken back to the lab and dried to obtain dry biomass by plots. Total biomass (TB) was obtained by summing above- and below-ground biomass. Root to shoot ratio (RSR) was calculated based on dry weight of below- and above-ground biomass. Subsamples of litter and root were processed for determination of C and N concentrations [31].

### Plant and soil C and N measurements

Soil organic C and plant sample C were measured by the potassium dichromate oxidation-external heating method [32]. Carbon oxidation occurred in potassium dichromate solution and C content was obtained by subsequent titration. Total N was digested by concentrated sulfuric acid and determined by the Kjeldahl method [32]. Carbon to N ratio (C/N) was calculated based on C and N concentrations. Soil NH_4_^+^ and NO_3_^-^ (AN) were extracted by 2 mol L^-1^ potassium chloride solution produced by deionized water and determined by the colorimetric method [8]. Microbial biomass C and MBN were measured by the chloroform fumigation—potassium sulfate extraction method [33, 34]. After extraction, MBC and MBN were measured with a TOC-1020A analyzer (Elementar, Germany). The TOC-1020A analyzer was also used for measurement of WSOC after extraction with deionized water (10 g soil with 20 ml water, shaking at 25 ^o^C for 15 min) and filtration using 0.45 μm polytetrafluoroethylene filters [35]. Water-soluble organic N was obtained by calculating the difference between total soluble N and inorganic N.

### Statistical analyses

We conducted pairwise correlation analysis to examine the correlations among soil C and N properties both across and within understory vegetation types to examine differences in these correlations induced by understory vegetation types. We performed principal component analysis (PCA) to understand the overall pattern of soil C and N variation among plots. We conducted paired *t* tests to examine differences in soil C and N characteristics between forests with two different kinds of understory vegetation types. No transformations were conducted since data met the assumptions of ANOVA. Statistical analyses were conducted in JMP 9.0 (SAS Institute, Cary, NC, USA).

## Results

### Differences in functional traits between two understory vegetation types

Both litter and roots produced by graminoid vegetation had lower C/N relative to that produced by *Dicranopteris* (Table 1). In addition, *Dicranopteris* was higher in TB and RSR compared to graminoid (Table 1).

**Table 1:** Differences in functional traits (mean ± 1 s.e.) between two understory vegetation types. Significant results of paired *t* tests are in bold. Root C/N, root C to N ratio; Litter C/N, litter C to N ratio. TB, total biomass, g m^-2^; RSR, root to shoot ratio.

Variables	*Dicranopteris*	Graminoid	*t* ratio	*P*
Root C/N	**36.02±0.49**	**33.43±0.42**	**-5.27**	**0.0008**
Litter C/N	**40.65±0.48**	**33.55±0.39**	**-7.33**	**<0.0001**
TB	250.10±4.69	240.28±4.29	**-2.96**	**0.0180**
RSR	**1.10±0.01**	**1.04±0.02**	**-2.35**	**0.0468**

### Soil C and N correlations across understory vegetation types

**Fig 2:**
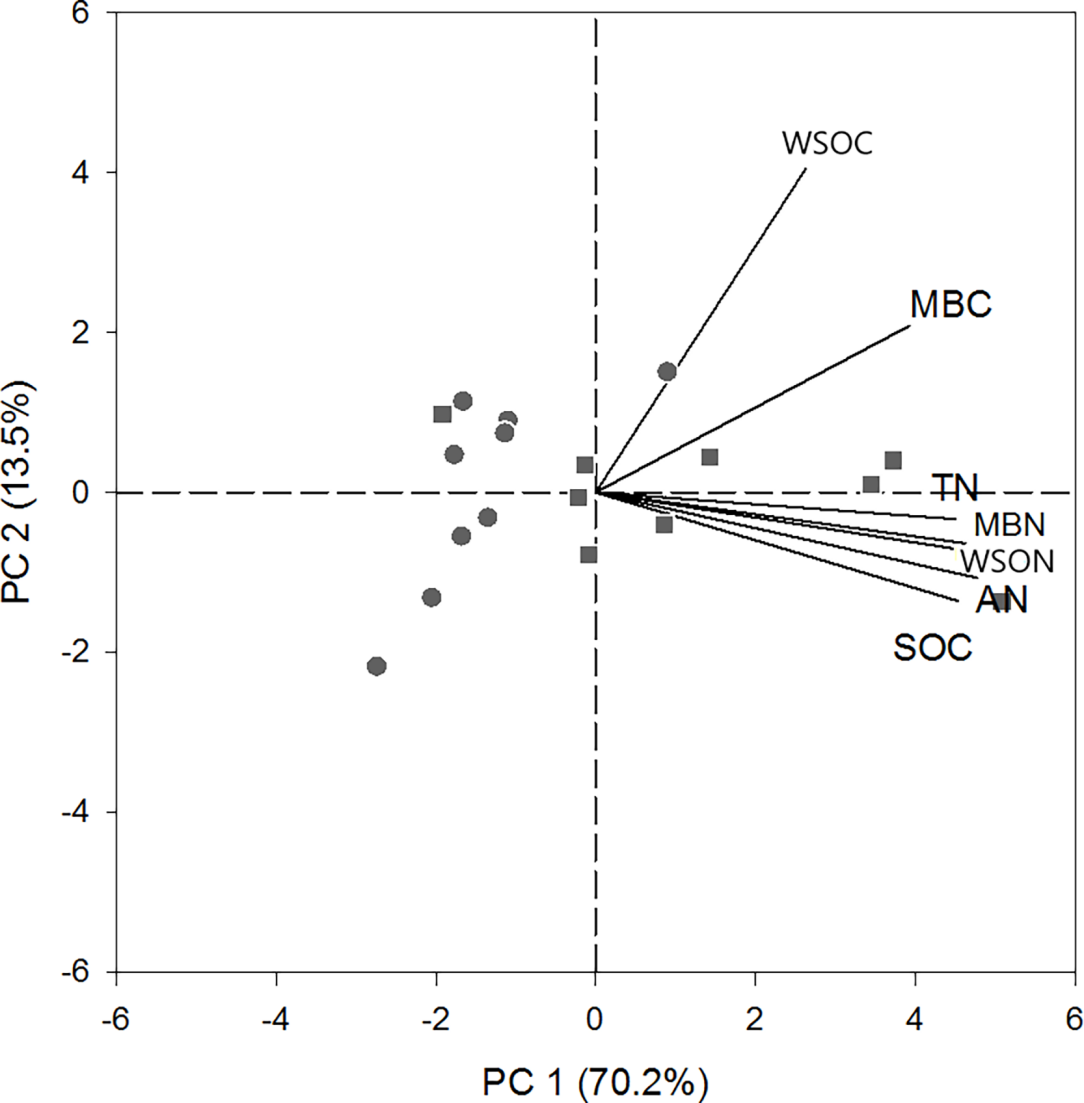
Principal components analysis (PCA) of C and N variables based on measurements from all plots. Filled circles, *Dicranopteris*; filled squares, graminoids.

Microbial biomass C was not correlated with SOC (Tables 2 and 3) but was only correlated with WSOC (Table 3). The PCA plot showed that, except WSOC, all the other variables contributed to the first principal component (Fig 2). The first principal component based on all variables explained 70.2% of the variations among all plots (Fig 2). The cumulative percentage of the first two components was 83.7% (Fig 2).

**Table 2:** Soil C and N variables (means ± 1 se) in plots with different understory vegetation types. SOC, soil organic carbon, g kg^-1^; MBC, microbial biomass carbon, mg kg^-1^; WSOC, water-soluble organic carbon, mg kg^-1^; TN, total nitrogen, mg kg^-1^; AN, available nitrogen, mg kg^-1^; MBN, microbial biomass nitrogen, mg kg^-1^; WSON, water-soluble organic nitrogen, mg kg^-1^. Statistical results are shown in Figs 3 and 4.

Type	Plot	SOC	MBC	WSOC	TN	AN	MBN	WSON
*Dicranopteris*	1	7.97±0.67	146.20±3.54	219.21±1.22	79.55±4.66	16.33±1.55	23.50±1.62	25.85±1.56
	3	10.21±1.61	190.22±5.23	300.38±3.08	84.41±2.03	22.01±1.27	33.11±1.35	40.25±1.75
	5	7.54±1.05	97.40±2.58	218.56±4.04	80.20±4.24	15.12±1.21	19.66±1.82	28.70±3.03
	7	2.55±0.54	259.38±4.62	234.80±3.38	75.79±3.28	15.38±1.10	24.27±1.71	20.53±1.78
	9	5.33±0.59	189.29±4.53	249.73±5.63	77.98±3.60	14.75±0.91	28.88±2.76	28.60±1.13
	11	5.13±0.06	96.42±3.31	147.14±5.00	73.69±2.11	15.00±1.08	17.35±1.85	32.81±1.24
	13	8.65±0.47	103.13±4.59	190.25±3.62	75.48±3.44	14.74±0.80	24.27±2.29	23.20±1.22
	15	4.42±0.16	179.82±2.40	226.36±3.43	72.10±1.16	10.32±1.19	22.74±1.28	37.15±1.56
	17	3.42±0.06	241.67±1.73	227.66±1.23	71.44±1.52	16.32±1.27	25.02±1.88	42.28±1.20
Graminoid	2	9.17±0.64	204.61±3.32	229.80±3.84	82.01±1.15	26.52±1.81	41.62±2.01	47.67±2.15
	4	16.84±1.00	267.90±4.04	243.64±1.40	96.45±2.83	53.83±1.39	67.74±0.94	63.23±1.70
	6	6.53±0.83	173.10±5.08	209.62±4.49	76.66±2.93	20.44±2.53	52.31±0.99	43.22±1.78
	8	9.27±1.21	223.56±5.81	257.60±3.84	87.65±2.72	29.79±0.47	40.79±1.30	43.03±1.32
	10	4.28±0.50	165.94±5.35	253.38±5.05	72.09±1.91	15.42±2.78	26.58±2.41	20.59±2.19
	12	6.62±0.92	177.52±3.62	234.42±3.83	86.80±2.32	17.36±2.28	32.72±1.85	38.00±1.23
	14	13.35±0.62	337.33±11.91	257.07±5.30	89.13±3.45	32.72±1.56	57.69±3.44	62.38±2.27
	16	8.29±0.63	199.81±1.85	244.29±2.00	87.89±2.07	15.46±2.33	19.67±3.98	40.72±2.16
	18	13.36±1.53	317.08±2.54	248.63±7.30	95.22±2.59	36.94±2.22	53.15±1.54	48.05±2.81

**Table 3:** Pairwise correlation coefficients (R) of soil carbon and nitrogen variables in the studied plots. SOC, soil organic carbon, g kg^-1^; MBC, microbial biomass carbon, mg kg^-1^; WSOC, water-soluble organic carbon, mg kg^-1^; TN, total nitrogen, mg kg^-1^; AN, available nitrogen, mg kg^-1^; MBN, microbial biomass nitrogen, mg kg^-1^; WSON, water-soluble organic nitrogen, mg kg^-1^.

Variables	MBC	WSOC	TN	AN	MBN	WSON
SOC	0.47	0.33	0.88**	0.88**	0.77**	0.75**
MBC		0.57*	0.57*	0.64**	0.68**	0.66**
WSOC			0.45	0.32	0.35	0.31
TN				0.81**	0.69**	0.70**
AN					0.89**	0.79**
MBN						0.81**

*, *P*<0.05

**, *P*<0.01.

### Understory vegetation effects on soil C and N characteristics

Correlation analysis of soil C and N variables under the two different vegetation types showed that both SOC and AN were correlated significantly with TN while WSOC was correlated with MBN for *Dicranopteris* plots (Table 4). For plots dominated by graminoid species, SOC, MBC, TN, AN, and WSON were correlated with each other while MBN was correlated with SOC, AN, and WSON (Table 4).

**Table 4:** Pairwise correlation coefficients (R) of soil carbon and nitrogen variables in plots dominated by *Dicranopteris* or graminoids. SOC, soil organic carbon, g kg^-1^; MBC, microbial biomass carbon, mg kg^-1^; WSOC, water-soluble organic carbon, mg kg^-1^; TN, total nitrogen, mg kg^-1^; AN, available nitrogen, mg kg^-1^; MBN, microbial biomass nitrogen, mg kg^-1^; WSON, water-soluble organic nitrogen, mg kg^-1^.

Vegetation type	Variables	MBC	WSOC	TN	AN	MBN	WSON
*Dicranopteris*	SOC	-0.56	0.26	0.76*	0.55	0.33	0.01
	MBC		0.56	-0.17	0.14	0.52	0.20
	WSOC			0.58	0.52	0.88**	0.34
	TN				0.72*	0.51	-0.11
	AN					0.60	0.23
	MBN						0.25
Graminoid	SOC	0.85**	0.34	0.86**	0.94**	0.79*	0.90**
	MBC		0.58	0.73*	0.70*	0.66	0.76*
	WSOC			0.35	0.27	0.01	0.11
	TN				0.74*	0.49	0.73*
	AN					0.86**	0.79*
	MBN						0.81**

*, *P*<0.05

**, *P*<0.01.

**Fig 3:**
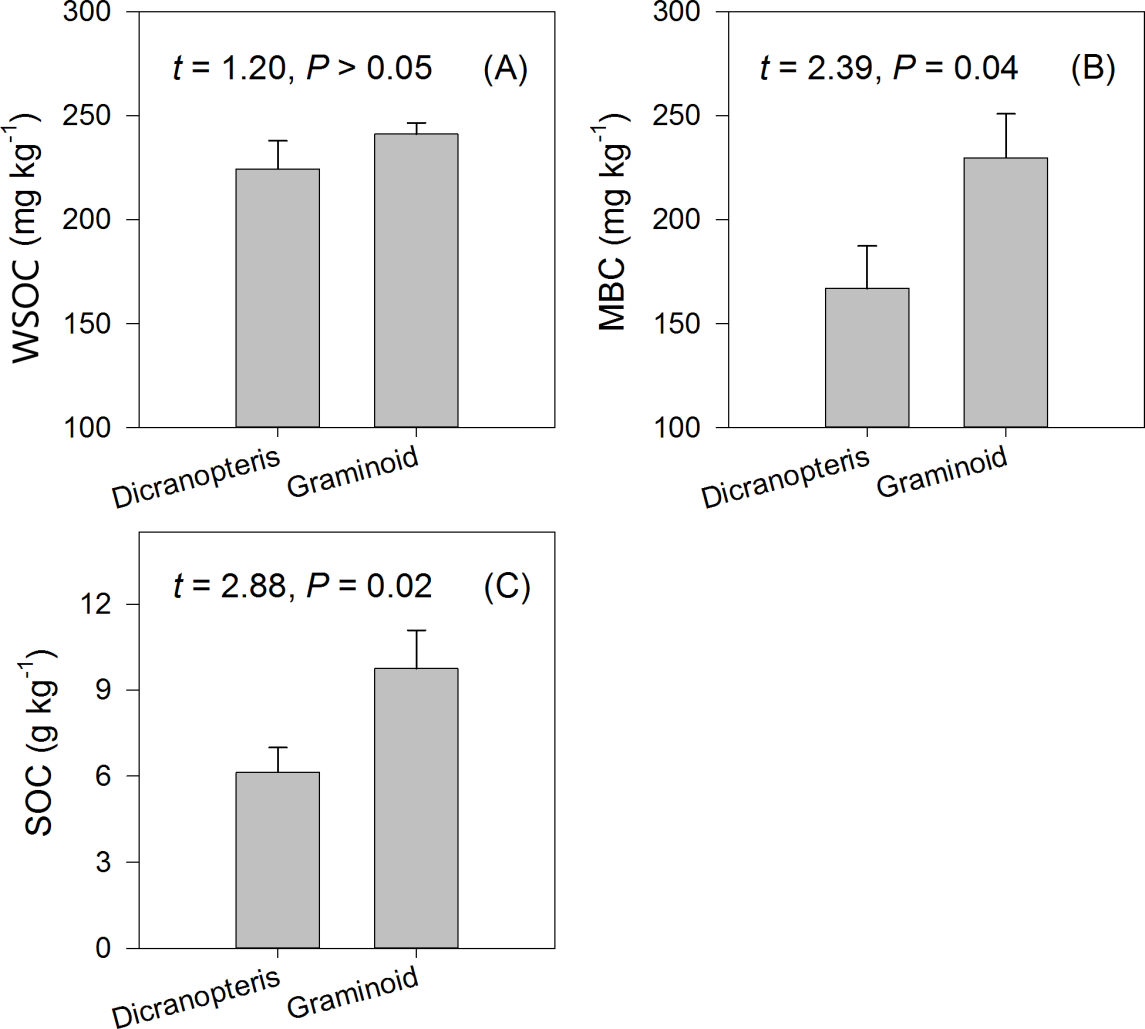
Dependence of soil C variables on understory vegetation types. Results of paired *t* tests are shown.

**Fig 4:**
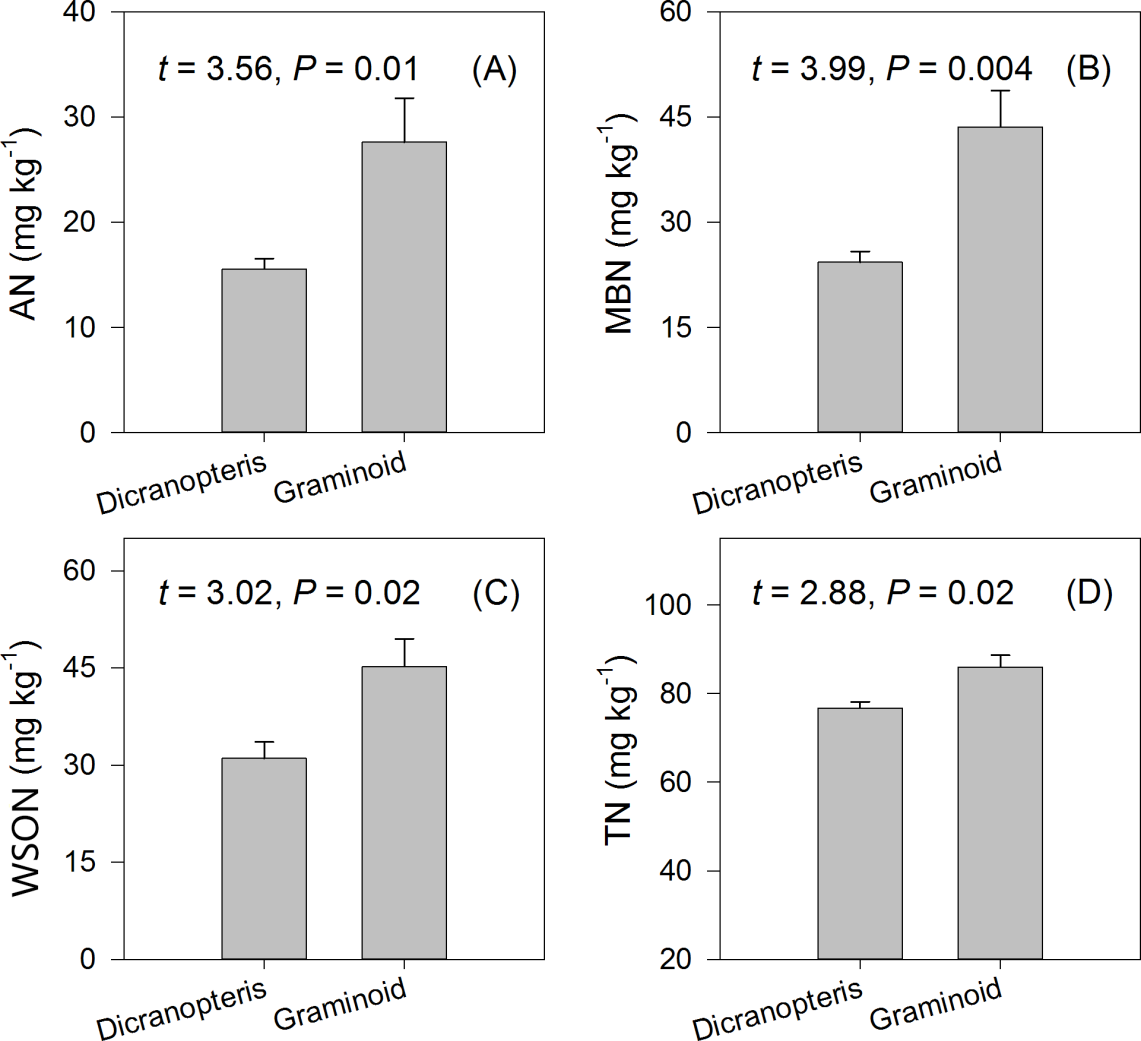
Dependence of soil N variables on understory vegetation types. Results of paired *t* tests are shown.

Except for WSOC (*t* = 1.20, *P* > 0.05), all the measured soil C and N variables were significantly higher in soils in plots dominated by graminoid species compared to those in plots dominated by *Dicranopteris* (Figs 3 and 4). Specifically, SOC, MBC, AN, MBN, and WSON were 58.8%, 37.5%, 77.5%, 79.3%, and 45.6% higher, respectively, in graminoid plots compared to *Dicranopteris* plots (Figs 3 and 4).

## Discussion

### Correlations between WSOC and MBC differ across and within understory vegetation types

Except WSOC, most of the variables correlated with each other across understory vegetation types [36]. Water soluble organic C has been commonly used as an indicator for microbial activities as it is readily available for microbes [37–39], which is consistent with the significant correlation between WSOC and MBC in this study (Table 3). When analyzed separately by understory vegetation, no correlations were observed between WSOC and MBC (Table 4). Although the study area had 18 plots, all plots were characterized by similar slope, tree diameter, canopy density and stand density (S1 Table), suggesting different effects of understory vegetation on MBC or WSOC. In addition, results of the PCA showed that WSOC was the only variable correlated positively with the second principal component, while all other variables positively correlated with the first component (Fig 2). Based on the spatial distribution of the data points with different treatments as well as MBC and WSOC, the results further indicated labile soil C variable (WSOC or MBC) was differently altered across understory vegetation types (Fig 3).

### Soil MBC and N variables increased by graminoids vegetation

Understory vegetation participates in the nutrient cycling of soil C and N process through several different ways. The graminoid vegetation has a fibrous root system, with higher decomposition rates compared with the rhizomes of *Dicranopteris*, which would potentially increase soil organic matter inputs [40, 41]. Additionally, due to differences in functional traits, understory vegetation types might alter litter decomposition rates and microbial activities by affecting soil moisture, temperature, and other environmental factors [42, 43]. Changes in the litter decomposition environment might alter litter decomposition rate and hence C and N releasing rate [44, 45]. Moreover, litter produced by different plant species or genotypes might differ in litter quality (e.g., lower C/N or higher N concentration) [46]. Both litter and root C/N of graminoids were significantly lower relative to *Dicranopteris* (Table 1). Higher litter quality (e.g., lower C to N ratio) might be associated with faster litter decomposition and hence faster nutrient releasing rate [44, 46], indicating graminoid vegetation might be more beneficial to enhancing nutrient cycling rates in areas it dominated. Due to differences in their functional traits, the quantity and quality of C and N released from decomposing litter and roots might differ among vegetation types [47, 48]. For example, Fu *et al*. [49] studied the effect of four shrub-grass types on SOC and TN in the Loess Plateau and found higher SOC in soils with vegetation characterized by higher aboveground biomass and underground root density. Chen *et al*. [50] proposed that soil N recycling rate and availability in soils with graminoid *Agropyron desertorum* was higher than that in soils with *Artemisia* plants due to differences in litter quality.

Except for WSOC, we found significantly increased SOC, MBC, AN, MBN, and TN in soils dominated by graminoids compared those with *Dicranopteris*, which is consistent with the higher litter and root quality of the graminoids (Table 1). In this study, the dominant species in plots with graminoids is *Paspalum thunbergii*, which is characterized by N fixing ability. The N-fixing ability might induce higher N levels in *P*. *thunbergii* soils [26], which would further increase the litter quality produced by *P*. *thunbergii*. Indeed, both litter and root N concentration were higher in graminoid areas, accompanied by higher soil N availability [44]. Stokdyk and Herrman [18] reported N-rich *Frangula alnus* leaf litter enhances soil N mineralization. Therefore, if there are no differences in soil abiotic factors, higher litter quality might be followed by faster C and N return rate and hence soil C and N availability. In addition, the positive effects of graminoids on soil C and N components were also consistent with the lower RSR of graminoids relative to *Dicranopteris*, indicating higher soil nutrient availabilities in these graminoid soils. Hence, the colonization of graminoid in plantations dominated by *Dicranopteris* should be promoted to improve the soil quality of aerially seeded *Pinus massoniana* plantations.

While soil microbial community and enzyme activity could have contributed to changes in soil C and N processes [51–53], which should be considered in further studies examining understory vegetation effects on soil C and N process. In addition, this study was conducted in areas with similar erosion levels before the colonization of both studied understory vegetation types. Areas with different erosion levels might also differ in understory vegetation types and soil element cycling. Future studies with manipulation levels of soil erosion and understory vegetation types would be necessary in understanding the effects of understory vegetation types on soil C and N cycling in aerially seeded plantations.

## Conclusion

Our results suggest that understory vegetation generated different effects on soil C and N processes. Graminoid understory vegetation with higher litter and root quality increased soil C and N components. Considering their significant contribution to atmospheric compositions and mitigations of global climate change, variation in soil C and N as affected by different understory vegetation should be considered in future studies, especially those in degraded areas where aerial seeding afforestation has been widely implemented with different understory vegetation types.

## Supporting information

S1 Table Description of the studied plots (DBH: Diameter at breast height; N: Tree number). (DOCX)
