# Supplementary material for: Impact of understory vegetation on soil carbon and nitrogen dynamic in aerially seeded Pinus massoniana plantations
Source: PLoS One. 2018 Jan 29;13(1):e0191952. doi: 10.1371/journal.pone.0191952 (PMC5788378; doi:10.1371/journal.pone.0191952)
Supplement: S1 Table — (DOCX) [file pone.0191952.s001.docx]

***Dicranopteris* and Graminoid Differ in Their Effects on Soil Carbon and Nitrogen Process in Aerially Seeded *Pinus massoniana* Plantations**

**Ping Pan ^1^, Fang Zhao ^2^, Jinkui Ning ^1^, Ling Zhang ^1 *^, Xunzhi Ouyang ^1 *^ and Hao Zang ^1^**

**1** College of Forestry, Jiangxi Agricultural University, Nanchang 330045, China, **2** College of Tourism and Territorial Resources, Jiujiang University, Jiujiang 332005, China

**Short title:** Understory Vegetation Impacts Soil C and N Process

* lingzhang09@126.com, lingzhang@jxau.edu.cn (L. Zhang) and oyxz_2003@hotmail.com (X. Ouyang)

**Supplementary table**

**Table S1. Descriptions of the studied plots (DBH: diameter at breast height; N: tree number).**

| Type | Plot | Altitude  (m) | Slope  (°) | DBH  (cm) | Canopy Density  (%) | Stand Density  (N ha^-1^) |
| --- | --- | --- | --- | --- | --- | --- |
| *Dicranopteris* | 1 | 187 | 25 | 7.8 | 0.6 | 2,479 |
|  | 3 | 163 | 30 | 8.9 | 0.4 | 1,898 |
|  | 5 | 188 | 26 | 9.2 | 0.4 | 1,900 |
|  | 7 | 177 | 20 | 7.7 | 0.5 | 2,197 |
|  | 9 | 214 | 24 | 8.6 | 0.4 | 1,874 |
|  | 11 | 210 | 28 | 8.0 | 0.5 | 2,542 |
|  | 13 | 286 | 25 | 9.4 | 0.3 | 1,325 |
|  | 15 | 292 | 27 | 9.1 | 0.5 | 2,310 |
|  | 17 | 205 | 30 | 8.8 | 0.4 | 1,946 |
|  | Means±se | 213±15 | 26±1 | 8.6±0.2 | 0.44±0.03 | 2052±125 |
| Graminoid | 2 | 185 | 22 | 7.0 | 0.7 | 2,598 |
|  | 4 | 169 | 33 | 9.9 | 0.4 | 1,299 |
|  | 6 | 186 | 25 | 8.6 | 0.5 | 2,345 |
|  | 8 | 174 | 21 | 7.8 | 0.6 | 2,497 |
|  | 10 | 219 | 23 | 8.3 | 0.5 | 2,273 |
|  | 12 | 216 | 31 | 9.6 | 0.5 | 1,879 |
|  | 14 | 227 | 26 | 8.3 | 0.4 | 1,800 |
|  | 16 | 220 | 22 | 8.4 | 0.5 | 2,073 |
|  | 18 | 218 | 27 | 9.7 | 0.4 | 1,841 |
|  | Means±se | 201±7 | 25±4 | 8.6±1.0 | 0.50±0.03 | 2067±136 |
